# Exploration of treatment in childhood Langerhans cell histiocytosis based on inflammatory and malignant symptoms: a pilot study

**DOI:** 10.1186/s13023-024-03151-8

**Published:** 2024-04-23

**Authors:** Hui-ling Lin, Qing-qing Zheng, Ru-lin Huang, Rong Hu, Xiao-dan Liu, Jia-yi Wang

**Affiliations:** 1 Department of Hematology, Guangzhou Women and Children’s Medical Center, Guangzhou Medical University, No.9 Jinsui Road, Tianhe District, Guangzhou, 510600 China; 2 Department of Pharmacy, Guangzhou Women and Children’s Medical Center, Guangzhou Medical University, Guangzhou, 510600 China

**Keywords:** Langerhans cell histiocytosis, Inflammatory, Malignant, Symptoms, Treatment, Children

## Abstract

**Background:**

Multisystem childhood Langerhans cell histiocytosis (LCH) patients, especially those with risk organ (RO) involved, had not been satisfactorily treated under the international traditional schemes as high incidences of reactivation with late sequelae were largely reported. Over years, we have observed that LCH patients with varied clinical symptoms responded differently to different drugs, suggesting the current grouping strategies based only on the number of organs involved might be inadequate. LCH has been defined as an inflammatory myeloid tumor, thus this study has innovatively divided LCH pediatric patients into inflammatory or malignant symptoms group, and given different intensity treatment regimens to different groups.

**Aim:**

This clinical study aimed to explore a more appropriate patient grouping system according to the LCH symptom presentations and examine the clinical outcomes of treatment strategies in different groups.

**Methods:**

According to the clinical manifestations, 37 cases of children were divided into Group A (only inflammatory symptoms) and Group B (malignant symptoms with or without inflammatory symptoms). Patients in Group A and B were initially treated with vindesine (VDS) and methylprednisolone (PSL), and VDS, PSL, pirarubicin (THP) and cyclophosphamide (CTX), respectively. Treatment responses were evaluated six weeks after the induction therapy in all patients, and the criteria were disease status and clinical scores of symptoms.

**Results:**

Pre- and post-treatment scores were 1.22 ± 0.547 and 0.00 ± 0.00 in Group A, and 14.79 ± 1.686 and 1.00 ± 1.563 in Group B, respectively. All patients had subsequentlly received maintenance therapy without progressive disease. The 4-year overall survival (OS) rate was 100% in both groups and the 4-year event-free survival (EFS) was 94.4% in Group A and 89.5% in Group B, respectively. There were no obvious adverse events (AE) in Group A, whereas the main AE in Group B were alopecia and non-lethal hematological toxicity.

**Conclusion:**

Stratification according to patients’ clinical symptoms, with low-intensity treatment for inflammatory symptoms (mild manifestations) and intensive treatment with multiple drugs for malignant symptoms (severe manifestations), is a positive exploration that simplifies stratification method, achieves good long-term remission of the disease, and obtains a higher survival rate and quality of life, which seemed to be more appropriate for LCH patients.

## Introduction

Langerhans cell histiocytosis (LCH) has been the most common histiocytic disease in pediatric population with an averaged incidence of five per million in children under 15 years old [1]. LCH could affect nearly all organs/systems, while the most commonly involved are the skeleton, skin, lymph nodes, liver, spleen, mucosa, respiratory and nervous system [2]. Unfortunately, over one third LCH patients would relapse or develop severe adverse sequelae [3], indicating sustained attention and follow-up are still in demand for better disease management. Notably, LCH may present a self-limiting or spontaneous regression in some patients, whereas a progressive or even lethal pattern in the others [3].

Histopathologically, LCH exhibits a dual nature characterized by both inflammatory and malignant features. Early LCH lesions typically display CD1a/CD207 + granulomas surrounded by a T cell-rich inflammatory response, accompanied by the presence of pro-inflammatory cytokines [4–7]. Additionally, a median of only 8% of pathological Langerhans cells (LCs) were identified within LCH lesions, whereas the majority of the lesions consisted of multiple inflammatory infiltrates [3]. Simultaneously, CD1A-positive LCH lesions also exhibit malignancy-associated characteristics such as invasion, evasion, metastasis, and resistance to cell death. The identification of the oncogenic driver mutation BRAF-V600E in over 60% of LCH lesions provides additional evidence supporting the malignant nature of LCH [8, 9]. The reclassification of LCH as an inflammatory myeloid neoplastic disorder by the World Health Organization (WHO) in 2021 is a reflection of its complex and multifaceted disease presentation [10, 11], although the exact pathogenesis remained uncovered.

 LCH I-IV schemes developed by the Histiocytosis Society (HS) and the JLSG-96 protocol from the Japan LCH Study Group (JLSG) are worldwide recognized therapeutic regimens for LCH diagnosis and treatment. LCH III and JLSG-96 classify LCH into single system (SS) or multi-system (MS) involvement based on the presence (RO +) or absence (RO-) of risk organs (RO) such as the liver, spleen, or blood system. The main distinction between these two therapeutic regimens lies in the core chemotherapy agents utilized. The LCH III protocol [12] primarily utilizes vinblastine (VBL) and prednisone (PRED), optionally supplemented with rescue therapy involving PRED, cyclophosphamide (CTX), doxorubicin (ADR), and methotrexate (MTX), contingent upon the achievement of complete remission or occurrence of reactivation. Treatment response is evaluated six weeks following induction therapy prior to subsequent one-year maintenance therapy. The JLSG-96 protocol [13], on the other hand, employs a more intensive induction chemotherapy regimen, comprising Arm A consisting of PRED, VCR, MTX, and Ara-C, with or without Arm B comprising PRED, CTX, ADR, and MTX, depending on the attainment of complete remission or occurrence of reactivation. Therapeutic efficacy is assessed six weeks post-induction therapy before commencing a half-year maintenance therapy.

It has been reported [12, 13] that SS-LCH patients could achieve favorable efficacy outcomes, with few reported mortality cases, and in some instances, spontaneous remission was observed even without treatment. The average overall survival (OS) and complete remission (CR) rate in risk organ-negative (RO-) multi-system (MS) LCH patients could reach 95% and above, respectively [14], whereas a lower event-free survival (EFS) of around 70% was observed. However, RO + patients have a lower CR rate of around 50–60% under either the LCH III or JLSG-96 protocol. Notably, OS and EFS of RO + MS-LCH patients treated with the JLSG-96 protocol (95% and 70%, respectively) were higher than those with the LCH III regimen (77% and 42%, respectively) [12-14]. However, as the accumulated doxorubicin dose could be as high as 210 mg/m^2^ in the JLSG-96 protocol, there is a higher risk of cardiotoxicity. Additionally, the JLSG-96 protocol could result in a longer duration of myelosuppression, leading to more severe infections, longer in-hospital stay, higher adverse event-related costs, and poorer tolerance, especially in SS and RO- MS-LCH patients. Although the LCH III protocol has fewer treatment-emergent adverse events, fewer RO + MS-LCH patients could achieve CR even with later rescue therapy compared to patients under JLSCG-96 protocols. In general, SS and RO- MS-LCH patients had better treatment outcomes compared to RO + MS-LCH patients under either the LCH III or JLSG-96 protocol, however, there is still an urgent need to improve therapeutic responses with less toxicity in RO + MS-LCH patients.

Theoretically, a novel grouping system and treatment protocol based on the severity of the clinical presentations, which indicate lower-intensity therapy for inflammatory symptoms, whereas higher-intensity therapy for malignant symptoms, seem to be more clinically appropriate and potentially beneficial to pediatric LCH patients.To achieve a higher survival rate and reduce treatment-related complications, we have developed a novel LCH grouping system and treatment protocol (LCH-GZWC-2018 (Wang)) based on patients’ clinical manifestations (inflammatory or malignant symptoms), followed with separate therapeutic regimens. This study aimed to observe the clinical efficacy and safety of LCH-GZWC-2018 (Wang) in pediatric Chinese LCH patients.

## Materials and methods

### Patients enrollment criteria and ethics statement

In total, 41 Chinese pediatric patients under 16 years old, who were newly diagnosed with LCH, were registered in our centre between March 2017 and May 2021. Diagnoses were confirmed by histopathologic findings in affected organs, which were positive for either S-100, or CD1a antigen, or both [12, 15–17]. This study was approved by the ethics committee of Guangzhou Women and Children’s Medical Center (ethics number: 2022040817032102) and all participants were fully consented the treatment protocol and signed the patient consensus form.

### Definition of inflammation and malignant symptoms

As LCH has dual-property in nature, this study attempts to categorize the various clinical manifestations of LCH into inflammatory or malignant symptoms. Rash, otorrhea, bone pain, eosinophilic granuloma of the long bones, and diarrhea [18], etc., which mostly characterized by eosinophilic granulomatous inflammatory background [14], are classified as inflammatory symptoms. Noninfectious persistent fever, gingival or palatal swelling and thrombocytopenia, etc., which mostly characterized by invasive malignancy characteristics, are classified as malignant symptoms. Detailed clinical manifestations of each symptoms were summarized in Table 1. Liver involvement, presented as hepatomegaly and jaundice, is mostly considered as RO + by LCH III and JLSG-96, whereas in our study it has been classified as an inflammatory symptom. The liver involvement histology suggested lobular Langerhans cell infiltrate with mixed inflammatory background [19]. Moreover, it is difficult to reverse the jaundice of sclerosing cholangitis, which may eventually progresses to cirrhosis even under intensive chemotherapy. Therefore, patients with liver involvement were grouped into Group A (regimen for inflammatory symptoms) in our protocol to avoid intensified chemotherapy thus to reduce the risks of severe hepatotoxicity without substantial clinical benefits.

**Table 1:** Inflammation symptoms and malignant symptoms

Symptoms	Involved organs and manifestations
Inflammatory symptoms	• Rash • Ear canal lesions: otorrhea or suspected hearing impairment • Bone damage: pain, limited mobility, hollow mass of the calvarium, eosinophilic granuloma of the long bones,scoliosis or vertebral compression fracture • Gastrointestinal symptoms: diarrhea, failure to thrive or evidence of malabsorption • Pulmonary symptoms: cough, shortness of breath • Liver symptoms: jaundice, low whiteness proteinemia, etc • Endocrine system symptoms: short stature, growth failure precocious or delayed puberty,hypothyroidism • Central nervous system symptoms: diabetes insipidus, neurodegenerative changes
Malignant symptoms	• Noninfectious persistent fever • Infiltrated symptoms: gingival or palatal swelling, splenomegaly, mass of body or organ, lymphadenopathy • Hematologic symptoms: leukopenia, thrombocytopenia, anemia not explained by other causes

### Grouping system

According to the symptoms presented, the patients were divided into two groups. Group A included cases only with inflammatory symptoms, while cases exhibiting malignant symptoms were classified as Group B. For cases presenting both inflammatory and malignant symptoms were also classified into Group B.

### Treatment protocols

**Fig. 1:**
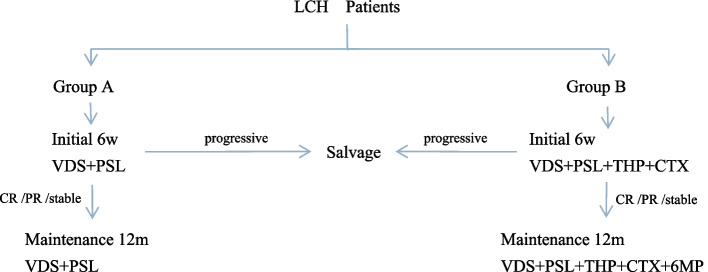
Treatment process of LCH-GZWC-2018 (Wang)

All patients received a six-week induction treatment, followed by a 12-month maintenance treatment. Group A was treated with vindesine (VDS) and methylprednisolone (PSL). Patients with bone lesions were included weight-bearing bone and functional bone, in addition to “special sites”, such as facial bones and Vertebra; Group B regimen was an intensive treatment combination of VDS, PSL, pirarubicin (THP) and CTX (see Table 2). According to the efficacy evaluation criteria of this study, as described in the following paragraph, if the patient was assessed to achieve CR, PR or stable, he/she could enter the maintenance phase of treatment after induction treatment. If the patient was defined as progressive, a salvage treatment would initiate (see Fig. 1). During the induction treatment, if some malignant symptoms sustained, vemurafenib (Vemur) was added, and if some inflammatory symptoms sustained, methotrexate (MTX) was added.

**Table 2:** Protocol of LCH-GZWC-2018 (Wang)

**Group A**			
**Initial treatment**(duration of 6 weeks)			
VDS	3 mg/m ^2^	IV	D1 D8 D15 D22 D29 D36
PSL	2 mg/kg	oral	D1-D28, D29-42 weekly tapering
**Maintenance treatment**(duration of 12 months)			
VDS	3 mg/m ^2^	IV	once per 3 weeks
PSL	2 mg/kg	oral	5 days per 3 weeks
**Group B**			
**Initial treatment**(duration of 6 weeks)			
VDS	3 mg/m ^2^	IV	D1 D8 D15 D22 D29 D36
PSL	2 mg/kg	oral	D1-D28, D29-42 weekly tapering
THP	20 mg/m ^2^	IV	D1 D15 D29
CTX	10 mg/kg	IV	D1-5 D15-19 D29-33
MTX^a^	25 mg/m ^2^	oral	once per week
Vemur#	15-20 mg/kg	oral	twice per day
**Maintenance treatment**(duration of 12 months)			
VDS	3 mg/m ^2^	IV	once per 3 weeks
PSL	2 mg/kg	oral	5 days per 3 weeks
THP	20 mg/m ^2^	IV	once per 6 weeks (W1, W7, W13,W19)
CTX	10 mg/kg	IV	once per 6 weeks (W4, W10, W16,W22)
6MP	50 mg/m ^2^	oral	once per day
MTX^a^	25 mg/m ^2^	oral	once per week
Vemur#	15-20 mg/kg	oral	twice per day

*VDS* Vindesine, *IV* Intravenous, *PSL* Methylprednisolone, *THP* Pirarubicin, *CTX* Cyclophosphamide, *MTX* Methotrexate, *Vemur* Vemurafenib, *6MP* 6mercaptopurine

^a^Additional use when inflammatory symptoms do not improve; #Additional use when tumor symptoms do not improve

### Efficacy evaluation criteria

Therapeutic efficacy was evaluated six weeks after the induction therapy in all patients, according to the disease status and clinical symptoms. Disease status was defined as the following: CR, all symptoms and signs subsided; PR, symptoms and signs improved, no new lesions; stable disease, some symptoms and signs persist without new lesions; progressive disease, progression of pre-existing symptoms and signs and/or emergence of new lesions (see Table 3). CR was termed as NAD; while PR, stable and progressive were termed as active disease (AD). Moreover, it has been reported recently that clinical score for disease activity could used to assess the therapeutic efficacy [20, 21], thus this study has referred to these reports to develop the clinical score of symptoms.The items of clinical score for each symptom are shown in Table 4. Comparing the clinical scores at the initial diagnosis and after the first six-week treatment to evaluate whether the clinical symptoms have improved after the application of the protocol.

**Table 3:** LCH disease status assessment

**Non-active disease (NAD)**	
Complete Response (CR)	All symptoms and signs subsided
**Active disease (AD)**	
Partial Response (PR)	Symptoms and signs improved, no new lesions
Stable disease	Some symptoms and signs persist without new lesions
Progressive disease^a^	Progression of pre-existing symptoms and signs and/or emergence of new lesions

^a^Progression of isolated bone damage is defined as appearance of new bone lesions or lesions in other organs

**Table 4:** Clinical score of symptoms

Symptoms	Manifestations	Scores
**Inflammatory symptoms**		
Rash (area of rash)	> 25%	2
	5 -25%	1
	< 5%	0
Ear canal lesions	Pus, itching, oozing	2
	Hearing abnormalities	1
	None	0
Bone damage	Pain, abnormal posture	1
	None	0
Gastrointestinal symptoms	Diarrhea	1
	None	0
Pulmonary symptoms	Need oxygen	2
	Symptomatic, no need for oxygen	1
	None	0
Liver symptoms (Child–Pugh classification)	Grade C	3
	Grade B	2
	Grade A	0
Endocrine system (excluding diabetes insipidus)	Replacement therapy	2
	No replacement therapy required	0
Central nervous system symptoms	Central nervous system mass or diabetes insipidus	3
	Decreased urine output	1
	Asymptomatic	0
**Malignant symptoms**		
Non-infectious fever	≥ 38 ℃	3
	None	0
Gingival or palatal swelling	Loose teeth, swollen gums	1
	None	0
Splenomegaly	Flat umbilicus under spleen ribs	3
	Spleen subcostal > 2 cm	2
	Spleen subcostal < 2 cm	1
	Spleen under rib	0
Mass of body or organ	Mediastinal mass	3
	No placeholder	0
Lymphadenopathy	Lymph nodes > 2 cm	2
	Lymph nodes < 2 cm	0
Hematologic symptoms	Hemoglobin < 110 g/L or platelet < 140 × 10 ^9^ /L	3
	normal	0

### Statistical analyses

Normally distributed parametric variables were expressed in mean ($\overline{{\text{x}} }$) and standard deviation (SD), while non-normally distributed parameters were in median and range. Clinical scores pre- and post-induction therapy were compared by paired-samples T test, the enumeration data were analyzed by the chi-square test, and the result of EFS was presented in Kaplan–Meier curve. A statistically significant difference was set at point-wise *p* value < 0.05. AEs were assessed according to CTCAE V5.0. The statistics were conducted in SPSS 21.0.

## Results

### Patient characteristics

Out of the 41 registered patients, 32 underwent BRAF immunohistochemical testing at the time of diagnosis, of which 20 were positive. Four of the 41 patients were excluded from the final analyses due to insufficient follow-up time (< 12 months). Consequently, 37 LCH pediatric patients were eligible for this study, including 18 patients with only inflammatory symptoms (Group A) and 19 patients with tumoral symptoms (original or progressed to; Group B). Patients were followed up for a median time of 38 months, ranging from 21.34 to 71.47 months.

Patient demographics and clinical characteristics are shown in Table 5. Both groups had more male patients than females (11 *vs* 7, and 10 *vs* 9, respectively). The median age at diagnosis was 3.3 and 1.0 years, in Groups A and B, respectively, suggesting a potentially younger onset age in malignant symptom group. The dominant manifestations in Groups A and B were bone damage (*n* = 18; 100%), and fever and hematologic symptoms (*n* = 19; 100%), respectively. In Group B, other common clinical manifestations were rash and liver symptoms (*n* = 17; 89.5%), splenomegaly (*n* = 16; 84.2%) and pulmonary symptoms (*n* = 12; 63.2%). In contrast, other symptoms including rash, ear canal lesions, gastrointestinal symptoms, liver symptoms and nervous system symptoms, were evenly distributed (1 patient (5.6%) each) in Group A.

**Table 5:** Patients' demographics and clinical characteristics

Characteristics	Group A	Group B
No. of patients	18	19
Age at diagnosis, y		
Median	3.3	1.0
Range	0.5—11.1	0.6—3.6
Gender		
Male	11(61.1%)	10(52.6%)
Female	7(38.9%)	9(47.4%)
Follow-up, m		
Median	38.08	38.93
Range	21.34—43.73	22.52—71.47
Inflammatory symptoms (no. of patients)		
Rash	1(5.6%)	17(89.5%)
Ear canal lesions	1(5.6%)	8(42.2%)
Bone damage	18(100%)	7(36.8%)
Gastrointestinal symptoms	1(5.6%)	5(26.3%)
Pulmonary symptoms	0	12(63.2%)
Liver symptoms	1(5.6%)	17(89.5%)
Endocrine system symptoms	0	1(5.3%)
Central nervous system symptoms	1(5.6%)	3(15.8%)
Malignant symptoms (no. of patients)		
Fever	0	19(100%)
Gingival or palatal swelling	0	1(5.3%)
Splenomegaly	0	16(84.2%)
Mass of body or organ	0	2(10.5%)
Lymphadenopathy	0	4(21.1%)
Hematologic symptoms	0	19(100%)

### Calculations of clinical scores of LCH symptoms

**Fig. 2:**
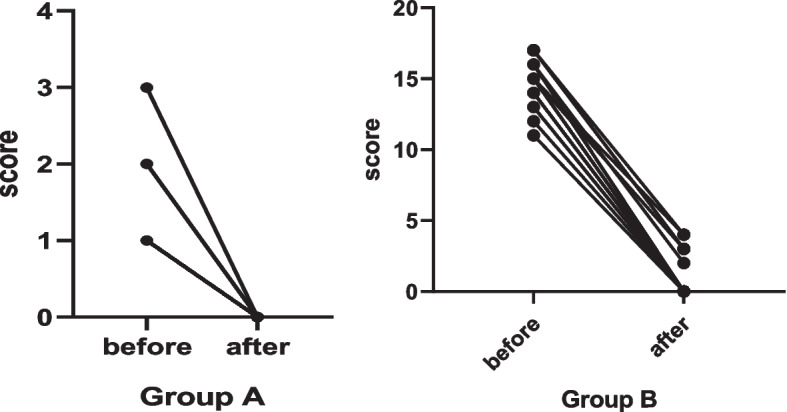
Clinical scores of symptoms before and after initial treatment

In pre- and post-induction therapy, Group A (*n* = 18) had clinical scores of 1.22 ± 0.547 and 0.00 ± 0.00, while Group B (*n* = 19) had clinical scores 14.79 ± 1 0.686 and 1.00 ± 1.563, respectively (see Fig. 2). The scores of two groups before and after treatment, analyzed by paired sample T test, were statistically significant (Table 6). This indicated that the clinical symptoms were significantly improved or subsided after initial treatment of this protocol.

**Table 6:** Evaluation of Treatment Effectiveness

	Group A	Group B
No. of patients	18	19
Clinical scores ($\overline{x }$± SD)		
At the primary diagnosis	1.22 ± 0.547	14.79 ± 1 .686
After the initial treatment	0.00 ± 0.00	1.00 ± 1.563
*p* value	< 0.01	< 0.01
Disease status		
NAD/better/stable	18 (100%)	19 (100%)
Progressive	0	0
Subsistence analysis		
4 -year EFS (%)	94.4%	89.5%
Event (diabetes insipidus)	1 (5.5%)	2 (10.5%)
Event’s time, M	4.54	18.64 / 20.25
4 -year OS (%)	100%	100%
Treatment-related adverse effects (Grade III or above of CTCAE 4.0)		
Hair loss	5 (27.8%)	18 (95%)
Fever	0	2 (10.5%)
Intestinal obstruction	1 (5.6%)	0
Hematological damage		
Anemia	0	12 (63.2%)
Thrombocytopenia	0	14 (73.7%)
Cardiac dysfunction	0	0
Hepatic dysfunction	0	0

### Four-year overall survival analysis

**Fig. 3:**
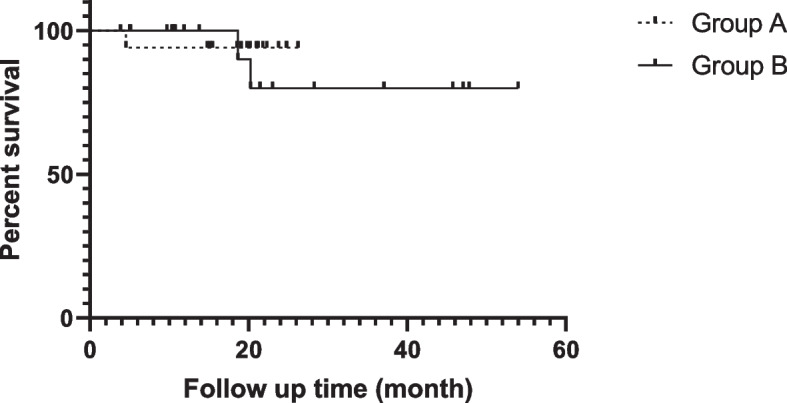
Four -year EFS analysis of patients in groups A and B

The OS of 4-year follow-up was 100% in both groups. After induction and maintenance therapy, the 4-year EFS of the two groups was 94.4% and 89.5% in Groups A and B, respectively (see Fig. 3). The main factor affecting EFS is the occurrence of diabetes insipidus. As shown in Table 6, Group A had one case (5.5%) of first-episode diabetes insipidus at follow-up, while Group B had two cases (10.5%), and the onset time of these events was 4.54, 18.64 and 20.25 months after treatment, respectively. At the time of diagnosis, these three patients were without polydipsia and normal urine specific gravity, and only one case underwent pituitary MR, whereas the other two failed to accomplish it for economic reasons. They were all able to survive for a long time with little impact on their lives after endocrine replacement therapy.

### Treatment-related adverse effects

Based on CTCAE 5.0, treatment-related AE of grade III and above were summarized in Table 6. In both Groups A and B, patients had well-tolerance under chemotherapy. One patient in Group A developed intestinal obstruction, and the post-hoc pharmacogennetic test reported that the patient carried a homozygous mutation of *CEP72* T/T, indicating a potentially high risk of VDS-induced peripheral neurotoxicity. The main AEs in Group B were alopecia and non-lethal hematological toxicity.

## Discussion

To our knowledge, this is the first study discussing a novel LCH grouping system and treatment protocol based on the severity of patients’ clinical manifestation of inflammatory or malignant symptoms. The overall 4-year OS and EFS have been the highest compared to the other published data in LCH cohorts, albeit with a relatively small patient number in this pilot study.

Guangzhou Women and Children’s Medical Center has started pediatric LCH diagnoses and treatment since the 1990s, with anually averaged number of 30 to 40 LCH cases. For years, we have observed an interesting phenomenon during the clinical practice of LCH treatment under LCH III or JLSG-96. When LCH is mainly manifested as inflammatory symptoms, such as systemic rash, multiple bone lesions, pus and exudate from the outer ear, long-term diarrhea, pneumonia, etc., the disease progresses sluggishly even with MS involvement, and the severity is normally of low-grade. Once malignant symptoms appear, such as long-term fever, splenomegaly, anemia, thrombocytopenia, and/or mediastinal mass, the disease would progress rapidly, severely or even fatally although fewer systems involved and the rash was normally mild.

During the 30 years of LCH treatment practices, we have also observed that LCH patients are with varied response to different drugs, suggesting the currently world-known grouping system and treatment protocol based only on the number or risk of organs involved might be insufficient. Based on the observed phenomena, we grouped patients according to their symptoms while scoring systems and disease status assessments have also been adjusted. In the other grouping systems, at least bi-lineages of the blood system should be affected when judged as blood system involvement, whereas in this study, patients showed continued anemia before or after treatment (excluding chemotherapy-induced or malnutritional reasons) even without leukopenia or thrombocytopenia were still judged as blood system involvement. When conducting liver evaluation, the method of judging the liver size (liver subcostal distance) was discarded, and the Child–Pugh liver function evaluation method was adopted. In terms of bone damage evaluation, not only the situation of special sites such as anterior bones, skull base bones, and vertebrae were considered, but also whether the affected bones are functional bones or load-bearing bones were taken into account in this protocol. The involvement of these bones could affect the patients’ quality of life, which is sometimes overlooked but highly recommended to be observed in previous classical LCH protocols. To reduce neurotoxiciy and cardiotoxicity, we used new alkaloid of VDS and THP, and reduced the dose of THP to 20 mg/m^2 ^to mitigating bone marrow suppression.

After grouping stratification according to the presentation of inflammatory or malignant symptoms, patients in the inflammatory symptom group who were treated with VDS + PSL only, have achieved good long-term EFS without adding vemurafenib, regardless of the presence of *BRAF* mutation. Only one patient with *CEP72* T/T genotype developed drug-related intestinal obstruction, which resolved within a short time after discontinuation of VDS. The patient was treated with reduced doses of VDS in the remaining phase of regimen without reoccurrence of intestinal obstruction. No significant adverse effects occurred in any of the remaining cases in Group A. This protocol demonstrates a novel treatment strategy with low chemotherapy toxicity, good tolerability, short hospitalization days and low cost.

Patients in the malignant symptom group, given intensive treatment with multiple drugs at the beginning, were able to control the disease progression rapidly, and all of them could reach NAD after six weeks of initial treatment, and then entered the maintenance phase of treatment, which also could achieve good long-term EFS. This protocol reduced the dose of anthracyclines, with a cumulative THP dose of only 160 mg/m^2^ (compared to the cumulative ADR of 210 mg/m^2^ in JLSG-96). Cardiac function and ECG were routinely monitored during the course of treatment, and no abnormalities were found. Most of the treatment-related adverse effects were alopecia and hematological toxicity. As the majority of patients in Group B have reached grade 4 (CTCAE 5.0) hematologic toxicity in the third week of this regimen, we effectively reduced the incidence of infectious complications by temporarily suspending the treatment. After the neutrophil count returned to 0.5 × 10^9^/L or above, the regimen was continued.

The time of first-episode diabetes insipidus in Group A was significantly earlier than in Group B, approximately 1 year earlier. This may be related to the involvement of the skull base bones in Group A patient, which needs to confirm by more cases, however, it reminds us that we should pay more attention to the urine volume and water intake of patients with skull base bones involvement during the follow-up. The event onset time of the two cases in Group B was after 18 months since treatment (6 months after drug withdrawal), which suggests a 1-year maintenance treatment might not be sufficient and longer duration of maintenance treatment should be considered in the future.

During the course of treatment, in both Groups A and B, we found that liver symptoms such as hepatomegaly, elevated liver enzymes and elevated bilirubin would be present for a long period in some patients. Nonetheless, the liver function assessment results remained at Grade A or B according to Child–Pugh classification, which did not lead to disease progression or affect their long-term survival. Additionally, we found glucocorticoid combined with low cytotoxic agents (e.g., VDS) could significantly slow down the progression of biliary sclerosis without intensive chemotherapy for hepatic symptoms only during LCH treatment. Our findings suggested that in pediatric LCH patients only presenting inflammatory symptoms, regardless of manifestation of MS or RO+ involvement, would still achieve reasonable disease control and/or good clinical outcomes with less intensive treatment of VDS + PSL.

## Conclusions

This research has proposed a novel grouping system and corresponding treatment protocol, which is an innovative exploration based on the previous LCH protocals developed by HS and JLSG. This research not only simplified the LCH grouping system but also achieved promising clinical results in a pilot study of 37 Chinese pediatirc LCH patients based on the newly developed therapeutic regimen. Larger validation cohorts are warranted in the future to evaluate the efficacy of this novel LCH treatment stratification method.

## Data Availability

The datasets used and/or analysed during the current study are available from the corresponding author on reasonable request.

## Abbreviations

CTX: Cyclophosphamide
EFS: Event-free survival
JLSG: Japan LCH study group
LCH: Langerhans cell histiocytosis
MS: Multisystem
NAD: Non-active disease
OS: Overall survival
PSL: Methylprednisolone
RO: Risk organ
SS: Single system
THP: Pirarubicin
VDS: Vindesine
